# Identifying radiomics signatures in body composition imaging for the prediction of outcome following pancreatic cancer resection

**DOI:** 10.3389/fonc.2023.1062937

**Published:** 2023-08-10

**Authors:** Gregory van der Kroft, Leonard Wee, Sander S. Rensen, Ralph Brecheisen, David P. J. van Dijk, Roman Eickhoff, Anjali A. Roeth, Florian T. Ulmer, Andre Dekker, Ulf P. Neumann, Steven W. M. Olde Damink

**Affiliations:** ^1^ Department of General, Gastrointestinal, Hepatobiliary and Transplant Surgery, RWTH Aachen University Hospital, European Surgical Center Aachen Maastricht (ESCAM), Aachen, Germany; ^2^ Department of Radiation Oncology (MAASTRO), GROW, School for Oncology and Developmental Biology, Maastricht University Medical Center, Maastricht, Netherlands; ^3^ Department of Surgery, Maastricht University Medical Center, European Surgical Center Aachen Maastricht (ESCAM), Maastricht, Netherlands; ^4^ NUTRIM School of Nutrition and Translational Research in Metabolism, Maastricht University, Maastricht, Netherlands

**Keywords:** pancreatic cancer, PDAC, body composition, wasting disorders, survival, radiomics

## Abstract

**Background:**

Computerized radiological image analysis (radiomics) enables the investigation of image-derived phenotypes by extracting large numbers of quantitative features. We hypothesized that radiomics features may contain prognostic information that enhances conventional body composition analysis. We aimed to investigate whether body composition-associated radiomics features hold additional value over conventional body composition analysis and clinical patient characteristics used to predict survival of pancreatic ductal adenocarcinoma (PDAC) patients.

**Methods:**

Computed tomography images of 304 patients undergoing elective pancreatic cancer resection were analysed. 2D radiomics features were extracted from skeletal muscle and subcutaneous and visceral adipose tissue (SAT and VAT) compartments from a single slice at the third lumbar vertebra. The study population was randomly split (80:20) into training and holdout subsets. Feature ranking with Least Absolute Shrinkage Selection Operator (LASSO) followed by multivariable stepwise Cox regression in 1000 bootstrapped re-samples of the training data was performed and tested on the holdout data. The fitted regression predictors were used as “scores” for a clinical (C-Score), body composition (B-Score), and radiomics (R-Score) model. To stratify patients into the highest 25% and lowest 25% risk of mortality compared to the middle 50%, the Harrell Concordance Index was used.

**Results:**

Based on LASSO and stepwise cox regression for overall survival, ASA ≥3 and age were the most important clinical variables and constituted the C-score, and VAT-index (VATI) was the most important body composition variable and constituted the B-score. Three radiomics features (SATI_original_shape2D_Perimeter, VATI_original_glszm_SmallAreaEmphasis, and VATI_original_firstorder_Maximum) emerged as the most frequent set of features and yielded an R-Score. Of the mean concordance indices of C-, B-, and R-scores, R-score performed best (0.61, 95% CI 0.56–0.65, p<0.001), followed by the C-score (0.59, 95% CI 0.55-0.63, p<0.001) and B-score (0.55, 95% CI 0.50–0.60, p=0.03). Kaplan-Meier projection revealed that C-, B, and R-scores showed a clear split in the survival curves in the training set, although none remained significant in the holdout set.

**Conclusion:**

It is feasible to implement a data-driven radiomics approach to body composition imaging. Radiomics features provided improved predictive performance compared to conventional body composition variables for the prediction of overall survival of PDAC patients undergoing primary resection.

## Introduction

The impact of cachexia, sarcopenia and myosteatosis on outcome following oncological surgery has been widely established (1–3). Reduced muscle function has been shown to be associated with myosteatosis, which is defined as increased inter- and intramyocellular fat stores (4, 5). It can be quantified by assessing skeletal muscle radiation attenuation (SM-RA) on Computed Tomography (CT)-scans (6–8). Both myosteatosis and high visceral adiposity have been reported to be associated with worse overall survival following a pancreatic oncological resection of Pancreatic ductal adenocarcinoma (PDAC) (9). Surgical resection is the only curative therapy available for the treatment of PDAC, but due to loco-regional advancement or metastasis, only 20% of pancreatic carcinomas are treatable by resection (10). Despite efforts to improve treatment efficacy, overall short- and long-term survival rates have remained poor over the past decades (11). This is partly due to wasting conditions such as cachexia and sarcopenia which occur in the vast majority of patients with pancreatic cancer (12, 13). Identifying patients who are predisposed to respond badly to surgical treatment based on body composition is of added value for personalized treatment strategies or pursuing non-surgical treatment alternatives.

Advances in CT image analysis have enabled identification of ‘tumor phenotypes’ by extracting large numbers of quantitative features from radiological images, i.e. the field currently known as radiomics. Radiomics features have been shown to provide prognostic value in predicting clinical outcomes of several tumor entities, including head and neck cancer and lung tumors (14–17). However, a recently published study of non-small cell lung carcinoma (NSCLC) patients undergoing chemotherapy treatment was unable to identify radiomics features that predict muscle loss (18). That study only investigated radiomics features of muscle tissue for the prediction of muscle loss. Chen et al, recently implemented a radiomics approach to the identification of sarcopenia in gastric cancer patients, and showed that radiomics measured sarcopenia outperformed conventional body composition analysis for survival and complication prediction (19). We hypothesized that additional phenotypic information related to body composition can be extracted from muscle, subcutaneous fat, and visceral fat compartments by a radiomics-based analysis of CT images in PDAC patients.

Our goal was to investigate whether radiomics-based body composition features can discriminate between patient groups with increased or decreased overall survival following curative resection for the treatment of PDAC. In addition, the performance of radiomics-based body composition analysis for the stratification of short versus long overall survival was compared to body composition variables obtained by conventional manual CT-scan analysis and established clinical patient characteristics.

## Methods

### Patients

All patients that had a resectable PDAC of the pancreatic head and were treated at Uniklinik Aachen (UKA) or Maastricht University Medical Center (MUMC), between 2010 and 2017 were eligible for inclusion. Patients were excluded from analysis on the basis of American Society of Anesthesiology (ASA) classification V, (severe liver cirrhosis with Child grade C, end-stage renal disease requiring dialysis, severe heart disease), New York Heart Association class IV, and/or chronic obstructive pulmonary disease (COPD) requiring (home)oxygen therapy and administration of neoadjuvant treatment. In addition, patients were excluded if CT-scans did not include the abdominal wall or when the interval between the time of the scan and surgery was greater than three months. Besides body composition, we evaluated age at the time of surgery and ASA-classification and BMI as clinical predictors (20, 21). Clinical data was acquired from a prospectively acquired database and retrospectively analysed. Ethical approval was obtained prior to this study from the local medical ethical board.

### CT body composition variables

Body composition was analysed using electronically stored venous or porto-venous intravenous contrast phase of abdominal CT-scans acquired during routine clinical practice. CT-scans were selected and analysed while blinded to the mortality outcomes by an experienced single investigator, trained using the gold standard of bodycomposition imaging methodology as described by Prado and Baracos et al, using Slice-O-matic software, version 5.0 (Tomovision, Montreal, QC, Canada) (22).

An overview of the different scanner parameters can be found in Appendix 1 of the **Supplemental Material**.

The third lumbar vertebra (L3) was used as a standard landmark to measure tissue cross-sectional area in cm^2^ as previously reported (13). In short, skeletal muscle area (SMA), visceral adipose tissue (VAT) area, and subcutaneous adipose tissue (SAT) area were quantified on CT images with manual segmentation using predefined Hounsfield Unit (HU) ranges (SM: -29 to 150 HU, VAT: -150 to -50 HU, and SAT: -190 to -30 HU) (23). SM, VAT, and SAT were corrected for stature to calculate the skeletal muscle index (SMI), VAT-index (VATI), and SAT-index (SATI) in cm^2^/m^2^, providing good estimates of total body SM, VAT, and SAT mass (23). Skeletal muscle radiation attenuation (SMRA) was assessed by calculating the average HU value of the total muscle area within the specified range of -29 to 150 HU.

Body composition greatly varies with gender. SM, VAT, SAT, SM-RA were therefore expressed as Z-scores. The Z-score is defined as the number of standard deviations each patient differs from the mean value of patients belonging to the same sex. The use of Z-scores facilitates comparison of the effects of body composition in heterogeneous patient cohorts, normalizing for the sex-based differences.

### Defining endpoints

The main clinical endpoint for the evaluation of survival following surgery was overall survival with a follow up of 5 years following surgery. In order to stratify patients into the best and worst performing 25% regarding overall survival compared to the middle 50%, Harrell Concordance Indexes (c-index) was used as the statistical discrimination metric. A body composition radiomics model was designed to evaluate whether bodycomposition radiomics features could predict best, middle and worst performing patients regarding overall survival, i.e. low, middle and high risk patients following pancreatic resection.

### Radiomics features optimization and model building

**Figure 1:**
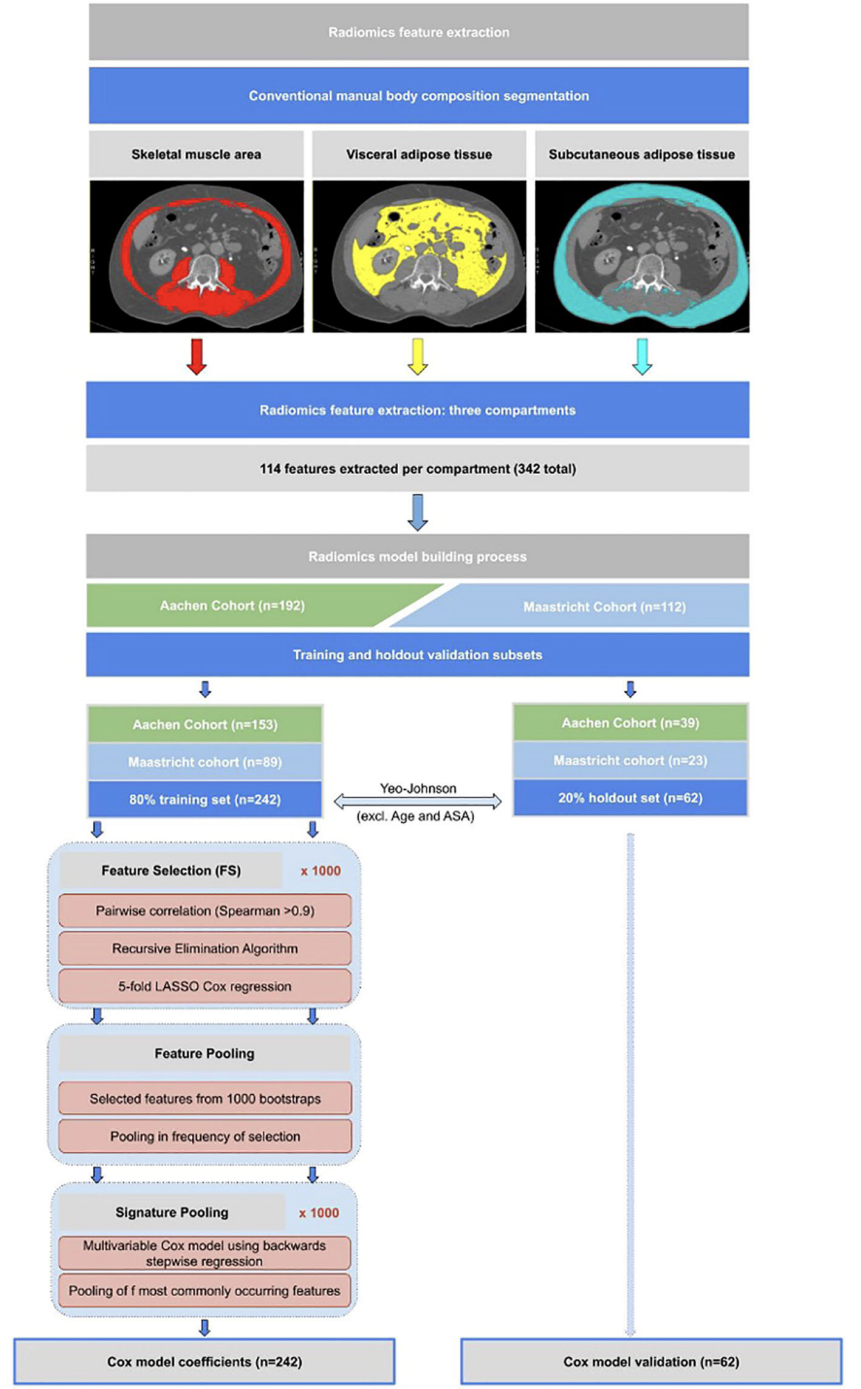
Flowchart showing methodological approach to radiomics feature extraction from CT scans, as well as the statistical model building methodology approach as described in the model building section.

In each segmented body composition region (SMA, VAT, and SAT), 114 individual radiomics features (342 in total) were automatically extracted using the open-source software library PyRadiomics 2.0.1 (24). The images were interpolated to a fixed 2mm grid during feature extraction to reduce unwanted variation. No digital image pre-processing filter was used. The model-building process consisted of the following key steps (illustrated schematically in **Figure 1**):

*Step 1: Training and holdout validation subsets*

The study population was split 80:20 into training and holdout sets, respectively, each maintaining the same proportion of Aachen and Maastricht patients as in the whole population. Survival outcome was calculated as time interval to death from date of surgery.

*Step 2: Feature value transformation*

A Yeo-Johnson transformation, followed by centering to a mean of zero and scaling to a standard deviation of one, was used to correct for highly skewed distributions of body composition and radiomics features in the training set (25). These same transformations, without recomputation, were applied directly on the holdout set.

*Step 3: Radiomics feature selection through regularization*

Due to the use of single CT-slices for analysis, thus no 3D imaging, we excluded all the 3D shape features from the feature selection. We first created 1000 random bootstrap samples (i.e. resampling from the training set with replacement) of the same size as the training set. These same 1000 resamples were kept fixed for feature selection (FS) and model development.

FS was applied only on the 342 radiomics features. For the clinical and body composition models, FS was omitted because these feature sets were already quite small (containing 3 and 4 predictors respectively as described in step 4).

In each bootstrap FS sample, we first calculated the radiomics features with very high pair-wise Spearman correlations (> 0.90). A recursive elimination algorithm was then used to remove the maximum number of redundant features. A 5-fold internally cross-validated LASSO (Least Absolute Shrinkage and Selection Operator) Cox regression was applied to the remaining features. The LASSO-selected individual features from each of the 1000 bootstrapped FS samples were pooled and ranked (from high to low) by its frequency of selection. We called these “surviving” features the *“feature pool”*. Since there is currently no universally accepted threshold for how frequently features ought to appear in a pool, we arbitrarily chose a cut-off frequency of 500. Features appearing less than 500 times out of 1000 bootstrap samples were assumed to be too sensitive to sampling and were therefore excluded from the stepwise regression step.

*Step 4: Signature pooling with stepwise Cox regression*

The same 1000 subsamples as described above were used to assemble a multivariable Cox model using backwards stepwise regression, with the objective to minimize the Akaike Information Criterion (26). This has the effect of testing different combinations of the most frequently LASSO-retained radiomics features. As before, we summed up the selected frequency of sets of features that appeared together, which we called the *“signature pool”*. Given that there is no consensus regarding which signature to choose from a number of alternatives, we decided to take the most-frequently appearing combination of features from the signature pool. The same process was used to build the clinical and body composition signatures. The clinical features selected by clinical experience *a priori* were – Age, BMI, ASA ≥3 and sex. The body composition features selected *a priori* were – SM-RA, SMI, VATI and SATI.

### Statistical analysis

The above mentioned feature selection and statistical analysis for survival was performed in R (20). Following Altman et al. (21), we computed a linear predictor, or “score”, from the sum of products of each feature with its coefficient. We computed the Harrell Concordance Index (C-index) for the training and holdout sets (23). The fitted regression predictors were used as “scores” for a clinical (C-Score), body composition (B-Score), and radiomics (R-Score) model. In order to stratify patients into the best and worst performing 25% regarding overall survival compared to the middle 50%, Harrell Concordance Indexes (c-index) was used as the statistical discrimination metric.

## Results

### Patient characteristics

Of the 425 patients included in the cohort, body composition analysis could be performed for 304 patients. In 52 cases, there was missing survival data, 14 patients were excluded on the basis of poor-quality CT scans, 25 were excluded due to scans not showing the abdominal wall, and 30 patients were excluded due to an interval greater than three months between the time of the scan and surgery. 53% of patients were male, with a mean age of 67.7 (SD 10.2) and mean BMI of 25.4 kg/m^2^ (SD 4.2). 42% of patients had a high ASA-score (≥3). 91.7% of patients underwent a Pylorus-Preserving Pancreatico Duodenectomy (PPPD) and 8.3% underwent a Whipple procedure (the same operation without preservation of the pylorus). Only PDAC cases (ductal adenocarcinoma) were included in the study. Ninety-day and two-year mortality rates were 10% and 45%, respectively. No differences were observed between in- and excluded cases regarding sex, age, ASA-score, or TNM classification (all p>0.10). Patient demographics across the training and validation cohorts are shown in **Table 1**.

**Table 1:** Demographics.

	Training-set	Validation-set	p-value
Age (mean)	66	68	0.21
Sex			
Male	114	28	0.86
Female	132	31	
BMI (mean)	25.6	25.9	0.48
ASA (mean)	2.41	2.47	0.39
Tumor stage			0.47
1A	3	1	
1B	12	1	
2A	46	17	
2B	161	36	
3	23	4	
SMI (mean)	44.7	46.9	0.90
SM-RA (mean)	34.4	34.2	0.91

Demographics of relevant study parameters across training- and validation-cohorts. P-value calculated using independent T-test for continuous variables and Chi-square test for binary variables.

### Feature selection and pooling results

Model building from feature selection to signature pooling (steps 1 to 4) was performed (see **Figure 1**) to select the most significant clinical-, body composition- and radiomics features and yielded a clinical score (C-score), body composition score (B-score), and a radiomics score (R-score).

### C-score

Age at surgery in years, ASA ≥ 3 and sex were used as prognostic clinical features for overall survival. The linear predictor equation in the Cox regression model was:


(Equation 1) C−Score=0.01353*(Age in years)+0.4087*(ASA≥3)+0.2803*(sex; male=1)

### B-score

**Figure 2:**
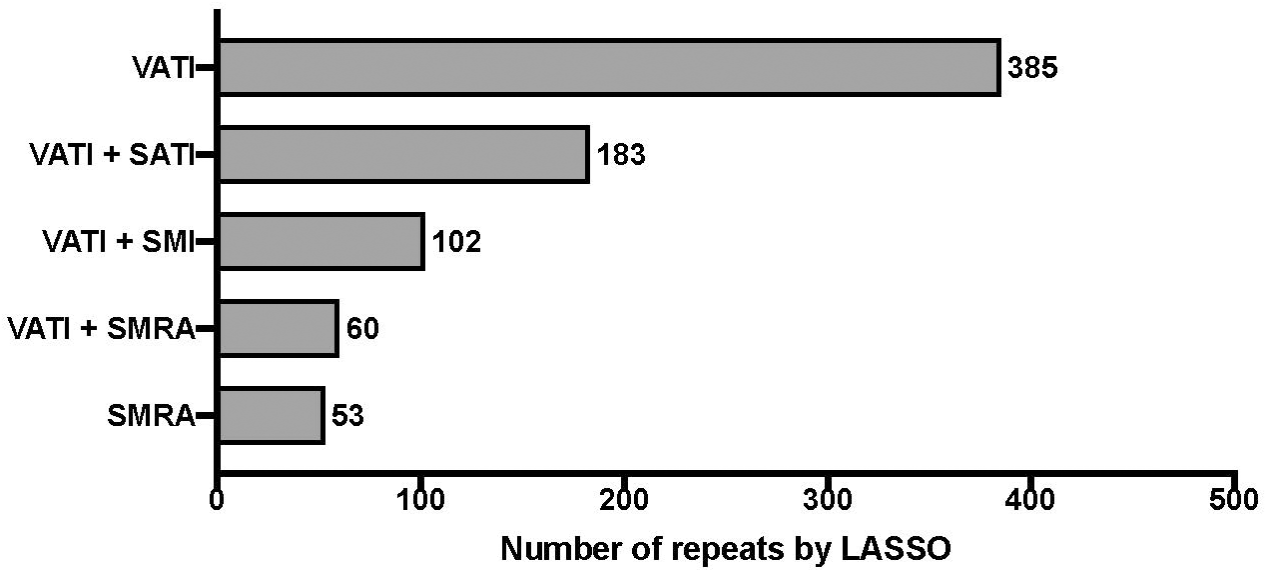
Relative importance of body composition variables of CT-scans on overall survival. “least absolute shrinkage and selection operator” (LASSO) penalized Cox regression with 5-fold cross-validation. Bootstrap resampling with univariate rejection and LASSO was repeated for a total of 1000 unique random number generator seeds. The frequency table, i.e. “feature pool”, of each body composition variable is shown; Visceral Adipose Tissue Index (VATI), Skeletal Muscle Radiation Attenuation (SM-RA), Skeletal Muscle Index (SMI), Subcutaneous Adipose Tissue Index (SATI).


The relative importance of the body composition variables SMI, SM-RA, VATI, and SATI for survival is shown in **Figure 2**. Increased VATI emerged as prognostic clinical feature for overall survival. The Cox model linear predictor was:

(Equation 2) B−Score =0.1988*(VATI)

### R-score

**Figure 3:**
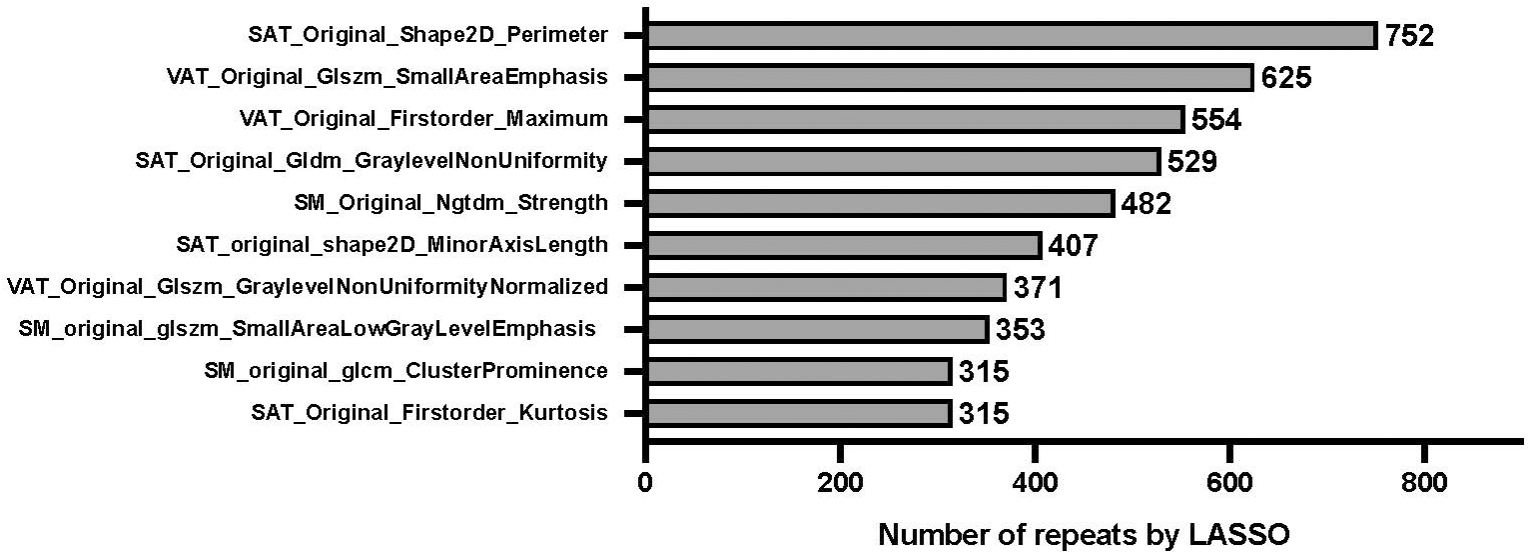
Relative importance of radiomics features on overall survival. “least absolute shrinkage and selection operator” (LASSO) penalized Cox regression with 5-fold cross-validation. Bootstrap resampling with univariate rejection and LASSO was repeated for a total of 1,000 unique random number generator seeds. The figure shows the frequency table of every radiomic feature that had a non-zero coefficient., with SAT_original_shape2D_Perimeter showing the highest number of repeats, i.e. being the most important radiomics feature. “VAT”, “SAT”, and “SM” in the formula refer to radiomics features extracted from visceral adipose tissue, subcutaneous adipose tissue, and skeletal muscle, respectively.

**Figure 4:**
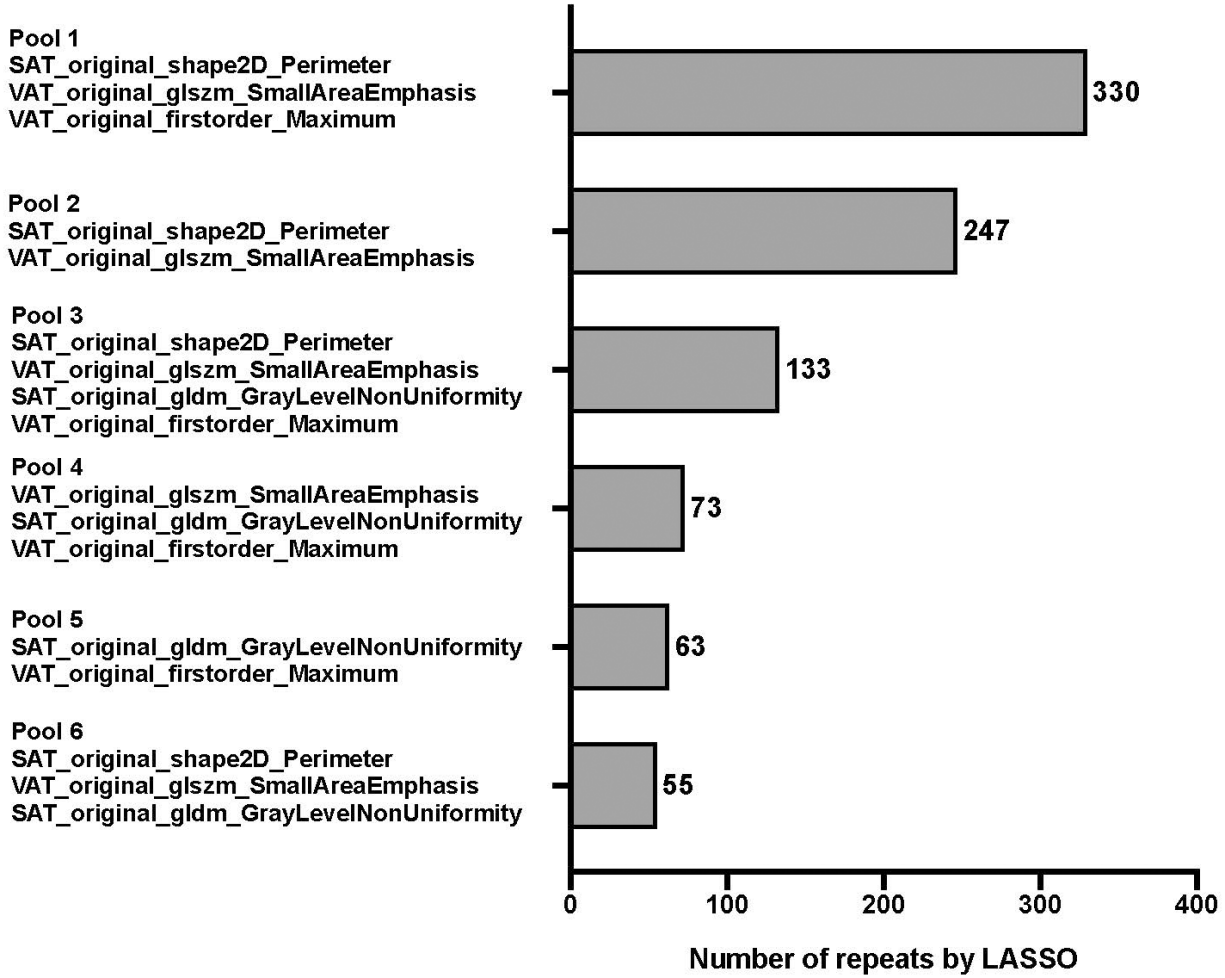
Frequency table for every combination of unique pooled features. Features were pooled by using 1,000 unique bootstrap samples consisting of a random 75% of the FS subset into a backwards stepwise Cox regression against survival time to assemble individual features into predictive signatures. The stepwise elimination criterion was the Akaike Information Criterion [1]. Frequency tables were compiled for every unique combination of features, ie signature. The pool with the highest number of repeats (pool 1) was finally selected for further modeling.


The results of *feature pooling* and *signature pooling* of the radiomics feature selection are shown in **Figure 3** and **Figure 4**, respectively. Four candidate radiomics features showed up more than 500 times and were selected from the LASSO-based feature pool. Various combinations of these four features were tested in the *signature pooling* step, and the most frequent set of features yielded an R-Score linear predictor comprising only 3 radiomics features:

(Equation 3) $$\begin{array}{c} R-\mathrm{Score}=\\ 0.3100*(\mathrm{SATI}\_\mathrm{original}\_\mathrm{shape}2D\_\mathrm{Perimeter})\\ -0.2302*(\mathrm{VATI}\_\mathrm{original}\_\mathrm{glszm}\_\mathrm{SmallAreaEmphasis})\\ +0.1353*(\mathrm{VATI}\_\mathrm{original}\_\mathrm{firstorder}\_\mathrm{Maximum}) \end{array}$$

To evaluate whether combined clinical, bodycomposition and radiomics models might outperform individual scores, we created combined CR (clinical & radiomics), CB (clinical and bodycomposition) and CBR (clinical, body composition and radiomics) scores.

### Modelling results for overall survival

In the training set, the mean Harrell C-indices for the overall survival time model were highest for the R-score (0.61, 95% CI 0.56 – 0.65, p<0.001), followed by the C-score (0.59, 95% CI 0.55 - 0.63, p<0.001) and B-score (0.55, 95% CI 0.50 – 0.60, p=0.03).

**Figure 5:**
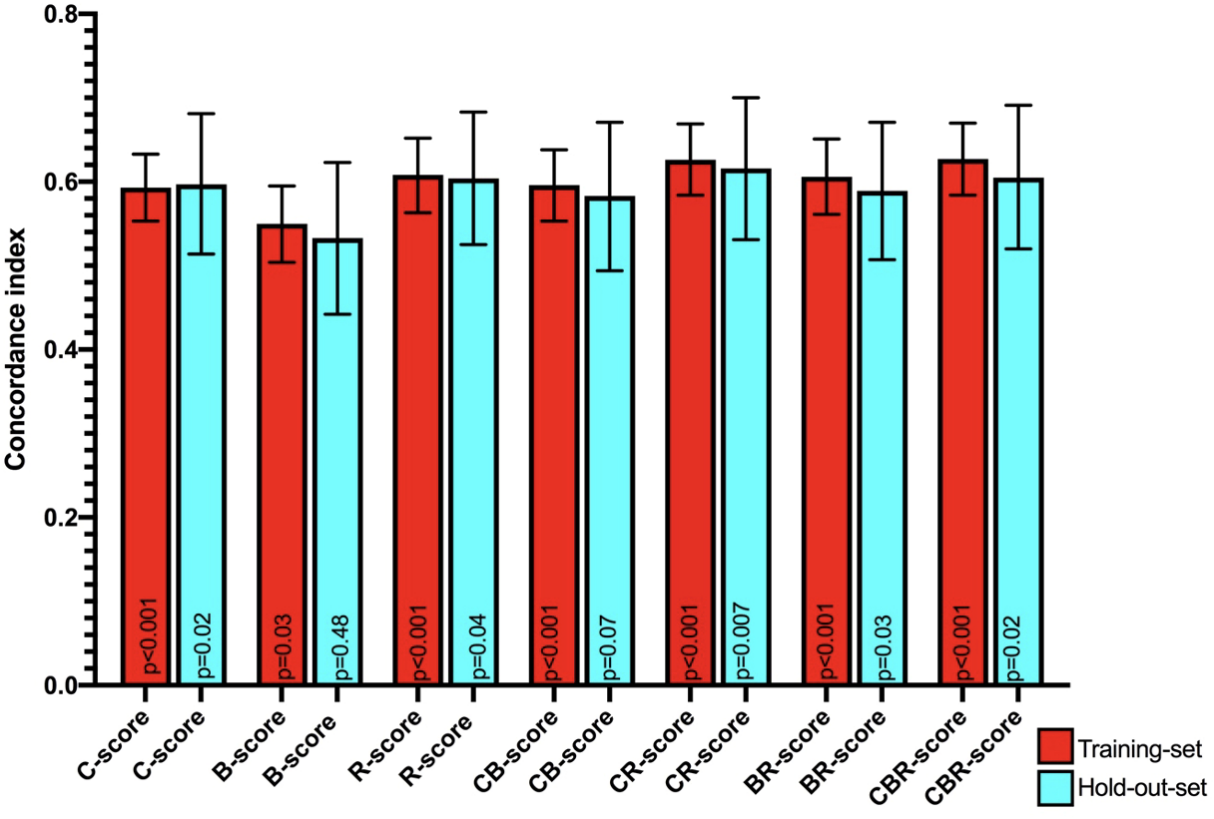
Mean Harrell concordance indexes of Clinical (C-score), body composition (B-score) and Radiomics (R-score) as well as combined clinical + body composition (CB-score), body composition + radiomics (BR-score) and clinical + body composition + radiomics (CBR-score) with corresponding 95% confidence intervals (error bars) and p-values from Log rank testing of both the training set (red) and hold-out-set (blue) are shown.

All three concordance indices were comparable in the holdout set compared to the training set: R-score: 0.60, 95% CI 0.53 – 0.68, p=0.04, C-score: 0.60, 95% CI 0.51 - 0.68, p=0.02, and B-score: 0.53, 95% CI 0.44 – 0.62, p=0.48, with the B-score not retaining significance in the hold out set (**Table 1** and **Figure 5**). Combined CR-score: 0.63, 95% CI 0.58 – 0.67, p<0.001, CB-score: 0.60, 95% CI 0.55 – 0.64, p<0.001 and CBR-score: 0.63, 95% CI 0.58 – 0.70, p<0.001, showed similar predictive value as individual scores, which were mostly reproduceable in the hold-out set CR-score: 0.62, 95% CI 0.53 – 0.70, p:0.007, CB-score: 0.58, 95% CI 0.49 – 0.67, p=0.07 and CBR-score: 0.61, 95% CI 0.52 – 0.69, p=0.02 and were therefore not plotted in the survival curves (**Figure 5**).

**Figure 6:**
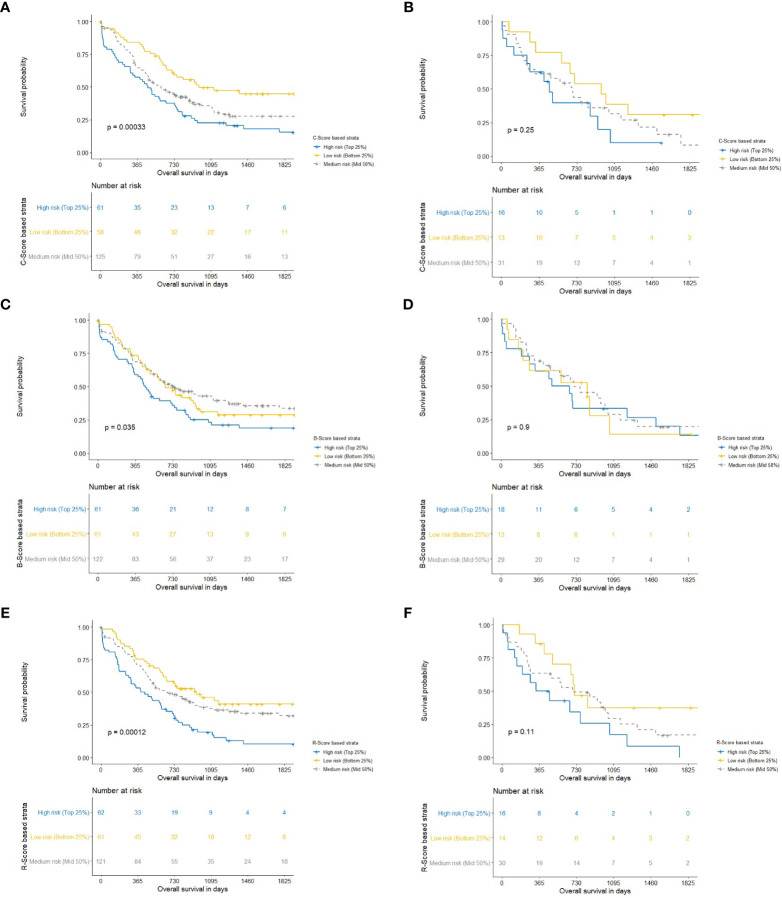
Kaplan-Meier charts with corresponding P-values for clinical (C-Score) for overall survival following pancreatic head resection for the treatment of PDAC. Blue, yellow and gray lines constitute the highest 25-, lowest 25-, and middle 50 percentages of risk. We computed the Harrell Concordance index (C-index) for the training set **(A)** and hold-out set **(B)** (25). The fitted regression predictors were used as “scores” for a clinical (C-Score) model. In order to stratify patients into the highest 25% and lowest 25% risk of mortality compared to the middle 50%. A Harrell Concordance Index (c-index) was used as the statistical discrimination metric. Kaplan-Meier charts with corresponding P-values for body composition (B-Score) for overall survival following pancreatic head resection for the treatment of PDAC. Blue, yellow and gray lines constitute the highest 25-, lowest 25-, and middle 50 percentages of risk. We computed the Harrell Concordance index (C-index) for the training set **(C)** and hold-out set **(D)** (25). The fitted regression predictors were used as “scores” for a body composition (B-Score) model. In order to stratify patients into the highest 25% and lowest 25% risk of mortality compared to the middle 50%. A Harrell Concordance Index (c-index) was used as the statistical discrimination metric. Kaplan-Meier charts with corresponding P-values for radiomics (R-Score) for overall survival following pancreatic head resection for the treatment of PDAC. Blue, yellow and gray lines constitute the highest 25-, lowest 25-, and middle 50 percentages of risk. We computed the Harrell Concordance index (C-index) for the training set **(E)** and hold-out set **(F)** (25). The fitted regression predictors were used as “scores” for a radiomics (R-Score) model. In order to stratify patients into the highest 25% and lowest 25% risk of mortality compared to the middle 50%. A Harrell Concordance Index (c-index) was used as the statistical discrimination metric.

From the survival curves (**Figure 6**), it can be seen that C-, B-, and R-scores all produced survival plots which discriminated between patients with an improved and decreased overall survival (p<0.001, p=0.035, and p<0.001, respectively). C- and R-scores showed best discrimination between patients with high, medium, and low risk for worse overall survival. However, the splits of all scores could not be significantly reproduced in the holdout sets.

## Discussion

In this study, we showed that it is feasible to implement a data-driven radiomics approach to body composition imaging, and that radiomics features can be extracted which perform comparably to conventional body composition variables for the prediction of overall survival of PDAC patients undergoing primary resection. Our data additionally indicate that VAT, SM, and SAT all contain radiomics features with potential predictive information for overall survival.

CT-based analysis of body composition has been validated for the quantification of whole-body muscle mass. This method has become increasingly popular to investigate the association between muscle mass, visceral adipose tissue mass, subcutaneous adipose tissue mass, and patient survival and/or response to treatment (22, 24, 27). Combined high visceral adipose tissue mass and low muscle mass have been shown to be associated with increased postoperative morbidity and mortality following oncological pancreatic resection (28). More recently, it has become evident that SM-RA, a radiological marker of myosteatosis, may be more indicative of wasting and decline of general condition and mortality in certain tumor entities, including pancreatic cancer (7, 29–31). In the current cohort, however, visceral adiposity was the most important conventional body composition variable associated with overall survival following pancreatic resection. In addition, the radiomics feature signature pool with the highest association with overall survival included features from the visceral and subcutaneous adipose tissue compartments. Although only the highest ranking radiomics features could be selected for cox regression analysis, the runner-up feature pool did contain radiomics features from the skeletal muscle compartment. Thus, although our radiomics model did not contain radiomics features from skeletal muscle VAT, SAT, and SM compartments may all contain radiomics features that hold predictive information for overall survival. Interpreting the descriptive nature of the radiomics features in our work or evaluating the association with anatomical or biological changes is speculative. When evaluating the nature of the radiomics features in our feature pools, it becomes apparent that all radiomics features are associated with subcutaneous and visceral fat area, not with muscle area. Implying a higher association of outcome with fat areas than with skeletal muscle. When specifically evaluating the radiomics features in our highest performing feature pool (feature pool 1), the most concrete association we could interpret, is SAT_original_shape2D_Perimeter. This implies a relationship with the two dimentional surface area of the subcutaneous fat area. However, the remaining features, VATI_original_glszm_SmallAreaEmphasis VATI_original_firstorder_Maximum are more difficult to interpret. The former is related to the Gray Level Size Zone (GLSZM), which quantifies gray level zones in an image. A gray level zone is defined as a the number of connected voxels that share the same gray level intensity within the visceral fat area. VATI_original_firstorder_Maximum describes the first order intensity of the signal within the visceral fat area. How these features relate to biological variations is difficult to extrapolate based on the data within this work and warrants further investigation into the basis of biological variation of radiomics features within fat tissue.

Radiomics imaging analysis originated in tumor imaging studies, where it was shown that large numbers of qualitative and quantitative CT features, which cannot be easily interpreted by the unaided human eye, could supply additional information about tumor heterogeneity, thereby giving a non-invasive estimation of disease severity (14–16, 32). De Jong et al. recently investigated whether skeletal muscle radiomics features are different in patients who develop muscle loss and those who maintain their muscle function after chemotherapy in a NSCLC cohort (18). Their study used two timepoints, prior to and following chemotherapy, to investigate changes in muscle mass and did not identify any muscle radiomics features associated with loss of muscle mass during treatment. The above-mentioned study solely extracted radiomics features from skeletal muscle and did not investigate the association between body composition, radiomics, and outcome, or correct for sex specific body composition variation.

Our clinical model (C-score) included age at the time of surgery, ASA-classification, and sex, and our body composition model (B-score) corrected for sex specific differences in body composition by implementing Z-scores. Due to a limited cohort size, splitting the cohort into male and female subgroups for radiomics analysis would have greatly underpowered our radiomics analysis. It was therefore not possible to establish whether sex specific differences in body composition produce different, sex-specific, radiomics signatures. It is however noteworthy, that despite the inability to perform sex-specific radiomics analysis, the radiomics model performed better than the body composition model for prediction of overall survival. This may imply that radiomics signatures are less subject to sex-specific differences than conventional body composition analysis.

We implemented a purely data driven approach to feature selection and modeling. However, it is important to note that predictive models which use a large number of candidate image features are susceptible to an increased risk of type I errors (32–34). This particularly holds true for studies with limited cohort sizes. Our cohort size implies a small holdout set. This could explain why all scores that showed a significant split of the Kaplan-Meier curves in the training set could not be reproduced in the holdout set. Although statistical significance was not achieved in the holdout set, we did observe a trend toward significance for the R-score. This could imply that our data may be underpowered to show the discriminatory value of radiomics for overall survival in the holdout set. The limited sample size additionally ruled out including all relevant radiomics features in our time-to-event model. Only one signature pool, containing three features, could be used for the time-to-event model for overall survival. This may have led to an underestimation of the cumulative effect of all relevant features. We were not able to test for repeatability and reproducibility of extracted radiomics feature values due to the fact that we did not have any explicit test-retest sample or multiple expert observers annotating the body compartments independently. However, we are confident that our method of 1000-times replication sampling and nested 10 times repeated 5-fold cross-validation with testing in a holdout set implicitly averaged out most of the instabilities of radiomics features with respect to hospitals, scanners, and imaging protocols. It is also likely that this approach limits the degree of overfitting in the current cohort.

Although body composition (i.e. sarcopenia and visceral adiposity) has been shown to be associated with worse outcome following pancreatic resection, body composition analysis has not found its way into clinical oncological treatment algorithms yet. Our data indicate that it is possible to identify a group of patients at risk for worse outcome based solely on body composition using radiomics. Our cohort, however, did not include patients receiving neoadjuvant- or palliative chemotherapy. Further evaluation of radiomics body composition analysis for the purpose of the identification of body composition-associated risk in chemotherapy treatment groups would provide valuable insights for clinical decision making and for the development of future treatment strategies.

## Conclusions

We found that it is feasible to implement a data-driven radiomics approach to body composition imaging, and we were able to extract radiomics features which held improved predictive value compared to conventional body composition variables for the prediction of overall survival of PDAC patients undergoing primary pancreatic resection. Furthermore, our data shows that VAT, SAT, and SM compartments all contained radiomics features that hold predictive information for overall survival. To gain actionable insight, larger cohort studies are needed to further investigate the added value of radiomics for the prediction of outcome for cancer patients.

## Data availability statement

The datasets presented in this article are not readily available because of a retrospective dataset. Requests to access the datasets should be directed to g.vanderkroft@maastrichtuniversity.nl.

## Ethics statement

The studies involving human participants were reviewed and approved by Ethic Commission RWTH-aachen. Written informed consent for participation was not required for this study in accordance with the national legislation and the institutional requirements.

## Author contributions

GK: concept, study design, data analysis, writing. LW: data analysis and conceptualization. SR: writing, supervision. RB: data analysis. DD: supervision. RE: data acquisition. AR: data acquisition. FU: data acquisition. UN: supervision. SO: conceptualization, paper writing, supervision. All authors contributed to the article and approved the submitted version.
