# Supplementary figures and images for: Identifying radiomics signatures in body composition imaging for the prediction of outcome following pancreatic cancer resection
Source: Front Oncol. 2023 Aug 10;13:1062937. doi: 10.3389/fonc.2023.1062937 (PMC10449585; doi:10.3389/fonc.2023.1062937)

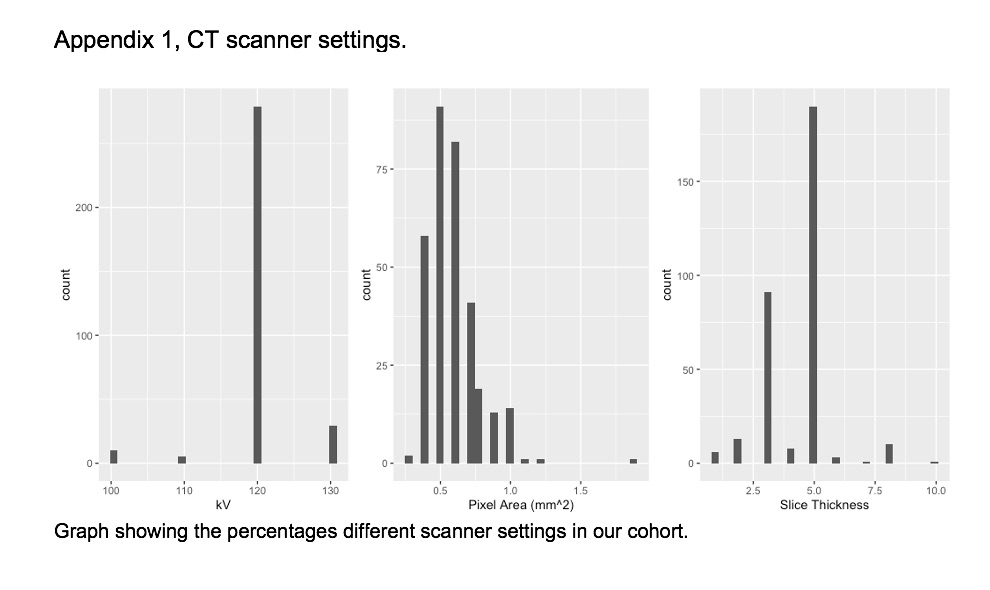

Supplement: Supplementary file 1 [file Image_1.jpg]
